# Salivary Inflammatory Biomarkers in Males With Nephrolithiasis Correlate With Periodontal Status: A Case‐Control Study

**DOI:** 10.1155/bmri/1619093

**Published:** 2026-06-13

**Authors:** Narjes Moneem Alhelfi, Ali Hadi Fahad, Salah M. Ibrahim, Sabah Qaysar Musa

**Affiliations:** ^1^ Department of Pedodontics, Orthodontics and Preventive, College of Dentistry, University of Kufa, Najaf, Iraq, uokufa.edu.iq; ^2^ Department of Oral Surgery, College of Dentistry, Kufa University, Najaf, Iraq, uokufa.edu.iq; ^3^ Department of Oral Diagnosis, College of Dentistry, Al-Muthanna University, As Samawah, Al-Muthanna, Iraq, uoalmuthana.edu.iq

**Keywords:** body mass index, IL-1*β*, IL-6, inflammation, MMP-8, nephrolithiasis, periodontal disease, salivary biomarkers, TNF-*α*

## Abstract

**Background:**

Systemic inflammation links periodontal disease (PD) and nephrolithiasis. Proinflammatory cytokines like IL‐6, IL‐8, TNF‐*α*, IL‐1*β*, and MMP‐8 are implicated in both conditions. This study investigates salivary levels of these biomarkers in young adult males with kidney stones and their association with periodontal status (CPITN) and body mass index (BMI).

**Methods:**

This case‐control study enrolled 109 males (25–35 years): 52 with kidney stones and 57 healthy controls. Unstimulated whole saliva was analyzed for IL‐6, IL‐8, TNF‐*α*, IL‐1*β*, and MMP‐8 via ELISA. Periodontal health was assessed using the community periodontal index of treatment needs (CPITN). Serum C‐reactive protein (CRP) and BMI were also measured.

**Results:**

The kidney stone group had significantly higher salivary IL‐6 (3.95 [2.0–5.2] pg/mL vs. 2.7 [1.5–4.0] pg/mL; adj. *p* = 0.042) and TNF‐*α* (11.99 [8.5–13.8] pg/mL vs. 9.5 [7.0–11.6] pg/mL; adj. *p* = 0.021) than controls. Differences in IL‐1*β* (adj. *p* = 0.051) and MMP‐8 (adj. *p* = 0.058) showed a consistent trend toward elevation but did not retain statistical significance after FDR correction. No significant difference was observed in salivary IL‐8 levels (adj. *p* = 0.120). Patients also showed elevated CRP (*p* = 0.010) and worse CPITN scores (*p* = 0.002). In the kidney stone group, salivary IL‐6, TNF‐*α*, and MMP‐8 positively correlated with CPITN scores (*p* < 0.01), whereas TNF‐*α* also correlated with BMI (*p* = 0.048).

**Conclusion:**

Elevated salivary inflammatory biomarkers (IL‐6 and TNF‐*α*) are significantly associated with kidney stones and periodontal status (CPITN) in young males, whereas IL‐1*β* and MMP‐8 showed a trend toward elevation. Salivary analysis may be a noninvasive tool for assessing systemic inflammation and identifying at‐risk individuals.

## 1. Introduction

PD and nephrolithiasis are common chronic diseases that have become a global health issue [1, 2]. Although they affect different organ systems, an association has been suggested, potentially mediated by systemic inflammation and oxidative stress [3–5]. In the United States, nephrolithiasis affects approximately 9% of the population, with a recurrence rate of up to 50% within 5 years, and its prevalence is increasing globally [6]. Periodontal disease is even more prevalent and is common in a large number of adult population around the world [7].

The oral cavity, as a reservoir of pathogenic bacteria and inflammatory mediators, can be involved in PD. Oral dysbiosis, an imbalance in the resident microbial community toward a more disease‐associated profile, is observed in PD [8, 9]. The pathogens associated with PD, such as *Porphyromonas gingivalis*, *Tannerella forsythia*, and *Treponema denticola*, can enter the bloodstream and contribute to a low‐grade systemic inflammatory response. This process may play a role in the pathogenesis of extraoral conditions, including chronic kidney disease (CKD) and nephrolithiasis [5].

At the microscale, calcium oxalate crystals formation, which represents the commonest type of kidney stone is capable to trigger NLRP3 inflammasome, which is an indispensable proportion of innate immune response [10, 11]. This activation leads to the generation of potent proinflammatory cytokines such as interleukin‐1 beta (IL‐1*β*) and therefore potentiating the inflammatory machinery [6]. Proinflammatory cytokines TNF‐*α* and IL‐6 were also reported to promote kidney stone formation and aggravate periodontitis by upregulating ROS [12–14]. Matrix metalloproteinase‐8 (MMP‐8) is a well‐established marker of periodontal tissue destruction and has been implicated in inflammation at other sites [15, 16].

BMI is also a variable that influences systemic inflammation. Adipose tissue is now considered an active endocrine organ and secretes several proinflammatory cytokines such as TNF‐*α*, IL‐6 [17]. Thus, obesity can lead to a chronic proinflammatory status, which could consequently predispose an individual to developing both PD and nephrolithiasis [18].

These linked pathways provide a valuable opportunity for noninvasive assessment of the systemic inflammatory burden through saliva biomarker profiling. Saliva is a rich source of biologically specific markers that represent both local and systemic health [19, 20]. Although a systemic inflammatory link between periodontal disease and nephrolithiasis has been suggested, the specific salivary inflammatory biomarker profile in patients with nephrolithiasis and its correlation with periodontal status remain poorly defined. Therefore, the present study is aimed at determining the levels of primary proinflammatory cytokines (IL‐6, IL‐8, TNF‐*α*, IL‐1*β*, and MMP‐8) in the saliva of young adult males with kidney stones and to investigate the relationship between these biomarkers, periodontal status (community periodontal index of treatment needs [CPITN]), and BMI. This specific demographic was chosen to minimize the potential confounding effects of hormonal fluctuations and age‐related comorbidities. CPITN was selected as a practical screening tool for periodontal status in this initial investigation, suitable for identifying treatment needs in a clinical setting.

## 2. Materials and Methods

### 2.1. Study Population

A case‐control study was conducted with male participants aged 25–35 years, recruited from the Al‐Sadr Hospital in Najaf, Iraq. The study was approved by the local ethics committee, and all participants provided written informed consent. The study group comprised individuals with a diagnosis of nephrolithiasis confirmed by a urologist based on clinical symptoms and imaging (ultrasonography or CT scans). Only patients with active stone disease were included. The control group consisted of systemically and periodontally healthy, age‐matched males.

### 2.2. Inclusion and Exclusion Criteria

#### 2.2.1. Inclusion Criteria

The inclusion criteria are as follows:

– Male participants aged 25–35 years.
– Diagnosis of nephrolithiasis confirmed by a urologist based on clinical symptoms and imaging (ultrasonography or CT scans).
– Active stone disease at the time of recruitment.
– Systemically and periodontally healthy, age‐matched males for the control group.

#### 2.2.2. Exclusion Criteria

The exclusion criteria are as follows:

– Current or former smokers.
– History of systemic diseases (e.g., diabetes mellitus, hypertension, cardiovascular disease, and autoimmune disorders).
– Use of anti‐inflammatory or antibiotic medications within the past 3 months.
– History of periodontal treatment (e.g., scaling, root planing, and surgery) within the past 6 months.
– Any other acute or chronic inflammatory conditions.

### 2.3. Sample Size

The total sample size was 109 participants. This was determined based on a power analysis to detect a medium effect size (*d* = 0.5) for the primary outcome variable, salivary IL‐6 levels, with an alpha of 0.05 and a power of 80%. This required a minimum of 51 participants per group. The study ultimately included 52 individuals in the kidney stone group and 57 in the control group.

### 2.4. Data Collection

For each participant, age was recorded, and height and weight were measured to calculate BMI. Periodontal health was assessed using the CPITN, as described by the World Health Organization [21, 22]. CPITN was chosen for its utility as a screening tool in a clinical setting, although it is acknowledged that it does not provide as detailed a measure of periodontal disease severity as a full‐mouth periodontal charting. CPITN scores were interpreted as follows: 0 = healthy; 1 = bleeding on probing; 2 = calculus; 3 = shallow pockets (4–5 mm); 4 = deep pockets (≥ 6 mm).

### 2.5. Saliva and Blood Sample Collection

Unstimulated whole saliva samples were collected between 9:00 and 11:00 AM to minimize the influence of circadian variation on biomarker levels. Participants refrained from eating, drinking, or chewing for at least1 h prior. Saliva was collected via the drooling method into sterile tubes for 10 min, following established protocols for sample integrity [23, 24]. Samples were centrifuged at 10,000 g for 15 min at 4°C, and the supernatant was stored at −70°C until analysis. All samples underwent a single freeze‐thaw cycle. Venous blood was collected for serum C‐reactive protein (CRP) measurement.

### 2.6. Biomarker Analysis

The enzyme‐linked immunosorbent assay (ELISA) method is validated for sensitivity and specificity in measuring salivary cytokines [25, 26]. Salivary concentrations of IL‐6, IL‐8, TNF‐*α*, IL‐1*β*, and MMP‐8 were determined using ELISA kits (Elabscience, Wuhan, China; R&D Systems, Minneapolis, Minnesota, United States) per the manufacturers′ instructions. The analytical characteristics for each biomarker were as follows:

– IL‐6: [LOD: 0.5 pg/mL; intra‐assay CV: < 8%; interassay CV: < 10%].
– IL‐8: [LOD: 1.0 pg/mL; intra‐assay CV: < 8%; interassay CV: < 10%].
– TNF‐*α*: [LOD: 0.8 pg/mL; intra‐assay CV: < 8%; interassay CV: < 10%].
– IL‐1*β*: [LOD: 0.3 pg/mL; intra‐assay CV: < 8%; interassay CV: < 10%].
– MMP‐8: [LOD: 0.1 ng/mL; intra‐assay CV: < 7%; interassay CV: < 9%].

All samples were analyzed in duplicate. Serum CRP levels were measured using a high‐sensitivity CRP assay, with a reference range of [typically < 3.0 mg/L for healthy individuals].

### 2.7. Statistical Analysis

Statistical analyses were conducted using SPSS (Version 23.0, IBM Corp., Armonk, New York, United States). The Shapiro–Wilk test was used to assess data normality for all variables. Data are reported as mean ± standard deviation (SD) for normally distributed variables and median (interquartile range) for nonnormally distributed variables. For group comparisons, either the independent samples *t*‐test or Mann–Whitney *U* test was performed. The Benjamini–Hochberg procedure for comparison of multiple biomarkers was performed, and significance set at *p* < 0.05. Pearson or Spearman correlation was used to run bivariate analyses of relationships between individual categorical and continuous variables. We conducted multivariable linear regression analysis to evaluate the independent associations of biomarkers and clinical variables, controlling for age and BMI. The regression analysis was performed with *p* value < 0.05 considered to be statistically significant.

## 3. Results

### 3.1. Baseline Characteristics

**Figure 1:**
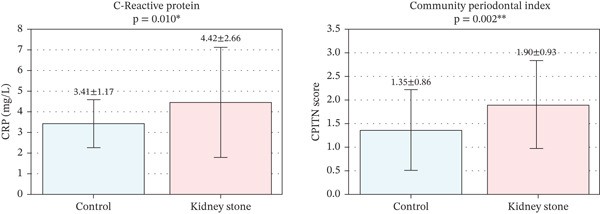
Baseline characteristics comparison: C‐reactive protein (CRP) and community periodontal index of treatment needs (CPITN). Bar charts comparing CRP levels and CPITN scores between control and kidney stone groups. Data are presented as mean ± SD. Statistical significance: ∗*p* < 0.05, ∗∗*p* < 0.01.

Baseline characteristics are presented in Table 1. There are no significant differences between study groups in mean age (*p* = 0.095) or BMI (*p* = 0.085). But patients in the kidney stone group had significantly higher serum CRP levels (4.42 ± 2.66 vs. 3.41 ± 1.17 mg/L, *p* = 0.010, Cohen^′^s d = 0.49) and CPITN scores (1.90 ± 0.93 vs. 1.35 ± 0.86, *p* = 0.002, Cohen^′^s d = 0.63) than controls (Figure 1).

**Table 1:** Baseline characteristics of study and control groups.

Parameter	Control group (*n* = 57)	Kidney stone group (*n* = 52)	*p* value	Cohen′s d
Age (years)	30.35 ± 2.70	31.25 ± 2.87	0.095	0.32
BMI (kg/m^2^)	23.98 ± 3.13	25.04 ± 3.21	0.085	0.33
CRP (mg/L)	3.41 ± 1.17	4.42 ± 2.66	0.010 ^∗^	0.49
CPITN score	1.35 ± 0.86	1.90 ± 0.93	0.002 ^∗∗^	0.63

*Note:* Data are presented as mean ± standard deviation (SD). Cohen′s d indicates the standardized effect size.

Abbreviations: BMI, body mass index; CPITN, community periodontal index of treatment needs; CRP, C‐reactive protein.

^∗^ *p* < 0.05.

^∗∗^ *p* < 0.01.

### 3.2. Salivary Biomarker Levels

**Figure 2:**
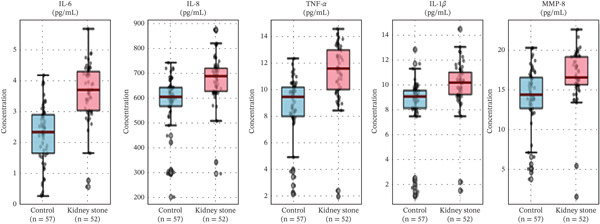
Comparison of salivary biomarker levels between kidney stone and control groups. Box‐and‐whisker plots showing the distribution of salivary biomarker concentrations in control (light blue) and kidney stone (light pink) groups. The central line represents the median, the box represents the interquartile range (IQR), and whiskers extend to 1.5× IQR. Outliers are shown as circles. This figure displays the median concentrations, consistent with the nonparametric analysis performed. Statistical significance: ∗*p* < 0.05, ∗∗*p* < 0.01.

Salivary concentrations of IL‐6, IL‐8, TNF‐*α*, IL‐1*β*, and MMP‐8 are presented in Table 2 and Figure 2. After FDR correction, salivary IL‐6 (adj. *p* = 0.042) and TNF‐*α* (adj. *p* = 0.021) were significantly higher in the kidney stone group compared with controls. Differences in IL‐1*β* (adj. *p* = 0.051) and MMP‐8 (adj. *p* = 0.058) showed consistent trends but did not retain statistical significance after correction for multiple comparisons.

**Table 2:** Salivary biomarker concentrations in study and control groups.

Biomarker	Control group (*n* = 57)	Kidney stone group (*n* = 52)	*p* value	Adj. p (FDR)	Cohen′s d
IL‐6 (pg/mL)	2.7 (1.5–4.0)	3.6 (2.0–5.2)	0.024 ^∗^	0.042 ^∗^	0.44
IL‐8 (pg/mL)	620 (500–730)	690 (550–880)	0.082	0.120	0.33
TNF‐*α* (pg/mL)	9.5 (7.0–11.6)	11.5 (8.5–13.8)	0.006 ^∗∗^	0.021 ^∗^	0.53
IL‐1*β* (pg/mL)	9.0 (7.0–11.0)	10.5 (8.0–13.0)	0.010 ^∗^	0.051	0.50
MMP‐8 (ng/mL)	15.5 (11.0–20.0)	18.0 (13.0–22.0)	0.033 ^∗^	0.058	0.41

*Note:* Data are presented as median (interquartile range). Adj. p (FDR) refers to *p* values adjusted via the Benjamini–Hochberg procedure to correct for multiple testing.

Abbreviations: IL, interleukin; MMP‐8, matrix metalloproteinase‐8; TNF‐*α*, tumor necrosis factor‐alpha.

^∗^ *p* < 0.05.

^∗∗^ *p* < 0.01.

### 3.3. Correlation and Multivariable Analysis

**Figure 3:**
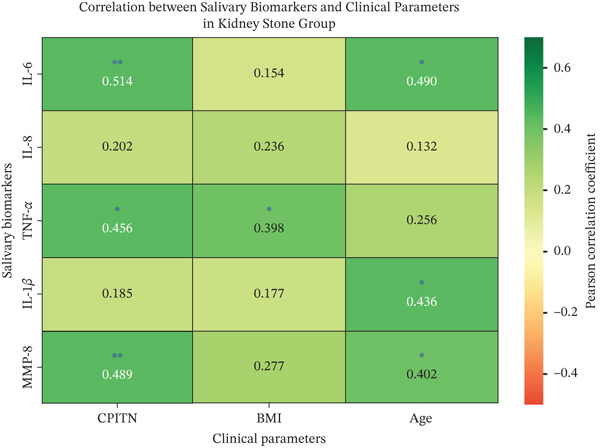
Correlation heatmap between salivary biomarkers and clinical parameters. Heatmap displaying Pearson correlation coefficients between salivary inflammatory biomarkers (IL‐6, IL‐8, TNF‐*α*, IL‐1*β*, and MMP‐8) and clinical parameters (CPITN, BMI, and age) in the kidney stone group. Color intensity indicates the strength of correlation, with green representing positive correlations. Statistical significance: ∗*p* < 0.05, ∗∗*p* < 0.01.

Univariate correlation analyses (Table 3, Figure 3) showed that salivary IL‐6, TNF‐*α*, and MMP‐8 were positively correlated with CPITN scores (*r* = 0.456–0.514, *p* < 0.05), and TNF‐*α* was correlated with BMI (*r* = 0.398, 95% CI [0.13, 0.61], *p* = 0.048). Multivariable linear regression analysis (Table 4), adjusting for age, BMI, and serum CRP, revealed that BMI was a significant independent predictor of salivary TNF‐*α* (*β* = 0.398, *p* = 0.048), whereas serum CRP was the strongest independent predictor for salivary IL‐6 (*β* = 0.412, *p* = 0.021) and MMP‐8 (*β* = 0.435, *p* = 0.012). CPITN remained a significant independent predictor for all three biomarkers (IL‐6: *β* = 0.380, *p* = 0.038; TNF‐*α*: *β* = 0.355, *p* = 0.042; MMP‐8: *β* = 0.410, *p* = 0.015).

**Table 3:** Detailed statistical parameters of univariate correlation analysis (including 95% CIs) between salivary biomarkers and clinical parameters in kidney stone group.

Biomarker	CPITN (*r*) [95% CI]	*p* value	BMI (*r*) [95% CI]	*p* value	Age (*r*) [95% CI]	*p* value
IL‐6	0.514 [0.28, 0.69]	0.004 ^∗∗^	0.154 [−0.13, 0.41]	0.416	0.490 [0.25, 0.68]	0.022 ^∗^
IL‐8	0.202 [−0.07, 0.45]	0.216	0.236 [−0.04, 0.47]	0.210	0.132 [−0.15, 0.39]	0.488
TNF‐*α*	0.456 [0.21, 0.65]	0.011 ^∗^	0.398 [0.13, 0.61]	0.04^8^ ^∗^	0.256 [−0.02, 0.50]	0.172
IL‐1*β*	0.185 [−0.10, 0.43]	0.328	0.177 [−0.11, 0.43]	0.350	0.436 [0.18, 0.64]	0.031 ^∗^
MMP‐8	0.489 [0.25, 0.67]	0.006 ^∗∗^	0.277 [0.01, 0.51]	0.138	0.402 [0.14, 0.61]	0.042 ^∗^

*Note:* r: Pearson correlation coefficient.

Abbreviations: 95% CI, 95% confidence interval; BMI, body mass index; CPITN, community periodontal index of treatment needs; IL, interleukin; MMP‐8, matrix metalloproteinase‐8; TNF‐*α*, tumor necrosis factor‐alpha.

^∗^ *p* < 0.05.

^∗∗^ *p* < 0.01.

**Table 4:** Multivariable linear regression between salivary biomarkers and clinical parameters in kidney stone group.

Biomarker	CPITN (*β*) [95% CI]	*p* value	BMI (*β*) [95% CI]	*p* value	CRP (*β*) [95% CI]	*p* value	Age (*β*) [95% CI]	*p* value
**IL-6**	0.380 [0.08, 0.65]	0.038 ^∗^	0.185 [−0.04, 0.41]	0.235	0.412 [0.15, 0.68]	0.021 ^∗^	0.120 [−0.10, 0.34]	0.354
**TNF-*α***	0.355 [0.06, 0.62]	0.042 ^∗^	0.398 [0.12, 0.67]	0.048 ^∗^	0.225 [−0.05, 0.50]	0.182	0.115 [−0.12, 0.35]	0.410
**MMP-8**	0.410 [0.15, 0.69]	0.015 ^∗^	0.280 [−0.02, 0.58]	0.124	0.435 [0.19, 0.68]	0.012 ^∗^	0.080 [−0.15, 0.31]	0.521

*Note*: *β*:Standardized regression coefficient.

Abbreviations: 95% CI, 95% confidence interval; BMI, body mass index; CPITN, community periodontal index of treatment needs; IL, interleukin; MMP‐8, matrix metalloproteinase‐8; TNF‐*α*, tumor necrosis factor‐alpha.

^∗^ *p* < 0.05.

## 4. Discussion

The clinical utility of salivary diagnostics is a subject of active research, with increasing evidence suggesting that saliva contains biologically specific markers reflecting systemic health [27, 28]. Recent comprehensive reviews have highlighted the vast potential of salivary biomarkers in diagnosing and monitoring a wide range of systemic conditions, including diabetes, cardiovascular diseases, and various cancers [29–32]. Our findings contribute to this body of evidence by demonstrating that salivary inflammatory cytokines are significantly elevated in young adult males with nephrolithiasis. However, these associations must be interpreted cautiously as potential markers of inflammatory burden rather than diagnostic tools. The cross‐sectional nature of this study does not permit any claims regarding diagnostic accuracy, predictive value, or clinical utility in routine screening.

The strong association between salivary inflammatory biomarkers and nephrolithiasis, independent of systemic metabolic status (BMI) and CRP, suggests that the oral cavity may reflect a distinct inflammatory compartment in stone‐forming patients. The biomarker‐periodontitis correlation highlights the importance of oral health in kidney stone patients, suggesting a need for interdisciplinary collaboration. This is supported by recent studies emphasizing the benefits of a collaborative approach between nephrologists and dental professionals, which has been shown to improve health outcomes and preparedness for kidney failure in patients with CKD [33–35]. Periodontal therapy itself has been shown to reduce systemic inflammation and improve vascular function, which could have a positive impact on kidney health [36–39], further longitudinal studies are required to determine if similar benefits occur in the context of renal stone prevention or recurrence.

The role of systemic inflammation in these conditions suggests that inflammatory pathways, such as NLRP3 inflammasome activation, may be involved in the pathophysiology of both periodontal disease and nephrolithiasis [40–42]. Although experimental studies have proposed potential anti‐inflammatory interventions, such as targeted nanoparticle‐based therapies [43], our study focuses exclusively on the association between markers and disease status. We emphasize that these mechanistic links remain speculative and require further investigation to differentiate between systemic inflammatory responses to stone formation and localized oral inflammatory processes.

The potential clinical application of salivary diagnostics is supported by recent advancements in point‐of‐care (POC) and microfluidic technologies, which enable the rapid, chair‐side analysis of inflammatory cytokines [44–46]. Although these platforms could theoretically facilitate patient assessment, their clinical application remains highly speculative and requires extensive validation [47]. Our cross‐sectional findings demonstrate associations between salivary inflammatory profiles and nephrolithiasis; however, the diagnostic or predictive utility of such biomarkers cannot be established from this study design. Before such technologies can be integrated into routine clinical workflows, rigorous evaluation of their diagnostic accuracy, cost‐effectiveness, and predictive value in prospective, multicenter trials is essential.

The oral microbiome is increasingly recognized as a key factor in the pathogenesis of systemic conditions, including nephrolithiasis. The emerging concept of a “gut‐kidney‐oral axis” suggests that microbial dysbiosis may drive systemic inflammation and the production of uremic toxins, thereby impacting both periodontal and renal health [48–53]. Our multivariable analysis identifies CPITN—a surrogate for periodontal inflammatory status—as an independent predictor of salivary inflammatory burden, even when adjusting for systemic markers like serum CRP. This aligns with the hypothesis that oral inflammatory status contributes to the systemic inflammatory milieu. The proposed therapeutic strategies such as probiotics and targeted antimicrobials [54, 55] are not supported by evidence in this context, and the causal relationship between oral dysbiosis and nephrolithiasis remains entirely speculative. Further mechanistic and longitudinal studies are required to determine whether oral microbial modulation can effectively influence renal stone pathogenesis.

Future research should focus on longitudinal studies to establish a causal relationship between salivary biomarkers, oral dysbiosis, and the development of nephrolithiasis. Larger, more diverse cohorts are needed to validate the findings of this study and to develop and validate multibiomarker panels for improved diagnostic accuracy. Furthermore, clinical trials are warranted to evaluate the efficacy of periodontal therapy and other interventions aimed at reducing systemic inflammation in preventing kidney stone recurrence. From a public health perspective, raising awareness about the oral‐systemic link and promoting interdisciplinary collaboration between dental and medical professionals are crucial steps toward improving the prevention and management of these prevalent chronic diseases.

## 5. Limitations

It is critical to acknowledge that our findings are subject to several methodological constraints. Case‐control design precludes causal inference; we cannot determine if elevated salivary biomarkers are a consequence or a contributing factor to nephrolithiasis. The use of CPITN as a periodontal screening index, although validated, does not provide a quantitative measurement of the total periodontal inflammatory surface area (PISA/PESA), which may have led to an underestimation of the oral inflammatory burden. Although we performed multivariable linear regression to adjust for age, BMI, and serum CRP, other potential confounders—such as oral hygiene practices, smoking, dietary habits, and medication usage—were not assessed. The absence of detailed renal clinical parameters (such as urinary calcium, oxalate, citrate, and uric acid levels) is a limitation. Finally, the relatively small sample size and homogenous demographic limit the generalizability of our results. Therefore, our conclusions remain preliminary, and the clinical application of these biomarkers must await validation in larger, multicenter, longitudinal studies.

## 6. Conclusion

This study found that young adult males with kidney stones had significantly higher levels of the salivary inflammatory biomarkers IL‐6 and TNF‐*α*. Differences in IL‐1*β* and MMP‐8 demonstrated a trend toward elevation but did not retain statistical significance after correction for multiple testing. A significant association was also observed between salivary TNF‐*α* levels and periodontal status (CPITN). These findings suggest a possible association between systemic inflammation, periodontal status, and nephrolithiasis in this population. However, due to the limitations of this study, including its cross‐sectional design and the use of a screening index for periodontal assessment, these results should be interpreted with caution. Further longitudinal research with more comprehensive periodontal assessments is needed to confirm and expand upon these findings.

**Nomenclature**
BMI: body mass index
CKD: chronic kidney disease
CPITN: community periodontal index of treatment needs
CRP: C‐reactive protein
ELISA: enzyme‐linked immunosorbent assay
IL‐1*β*
: interleukin‐1 beta
MMP‐8: matrix metalloproteinase‐8
NLRP3: NOD‐like receptor protein 3
PD: periodontal disease
POC: point‐of‐care
ROS: reactive oxygen species
TNF‐*α*
: tumor necrosis factor‐alpha

## Author Contributions

**Narjes Moneem Alhelfi:** conceptualization, data collection, sample analysis, writing—original draft. **Ali Hadi Fahad:** supervision, methodology, writing—review and editing. **Sabah Qaysar Musa:** data analysis, validation, writing—review and editing. **Salah M. Ibrahim:** project administration, supervision, and critically revised the manuscript.

## Funding

No funding was received for this manuscript.

## Ethics Statement

This study was approved by the Ethics Committee of Al‐Sadr Teaching Hospital, Najaf, Iraq (Reference Number: UK‐FD‐IRB‐2024‐103). All participants provided written informed consent prior to enrollment.

## Conflicts of Interest

The authors declare no conflicts of interest.

## Data Availability

The data that support the findings of this study are available from the corresponding author upon reasonable request.
